# Firearm injury—a preventable public health issue

**DOI:** 10.1016/S2468-2667(22)00233-X

**Published:** 2022-11-02

**Authors:** Jay Patel, Katherine Leach-Kemon, Gwenetta Curry, Mohsen Naghavi, Devi Sridhar

**Affiliations:** a Global Health Governance Programme, Usher Institute, University of Edinburgh, Edinburgh, UK; b Faculty of Medicine and Health, University of Leeds, Leeds, UK; c Institute for Health Metrics and Evaluation, School of Medicine, University of Washington, Seattle, USA

## Abstract

Firearm-related injury is a leading cause of death disproportionately affecting adolescents and young adults across the world, especially in the Americas. Little progress has been made over the past four decades, as inaction and the adoption of ineffective or unevidenced interventions have become commonplace. The COVID-19 pandemic reconfigured health systems towards prevention and harm reduction, sharpened public attention to the burden of preventable deaths, and inspired a fresh ambition of eliminating avertable deaths. In this Viewpoint, we argue that preventing firearm injury should garner bolder action in post-pandemic public health and we present a case for reducing the global burden of firearm injury supported by evidence and international examples. Crucially, we aim to guide policy making in directions that end the cycle of grief, anger, activism, deflection, and inaction and create more peaceful and fairer societies.

## Introduction

Without action, firearm-related injury will prevail as a post-pandemic epidemic that should not exist. In the past decade, violent, suicidal, and unintentional uses of firearms have claimed more than 2·75 million lives (95% CI 2 544 934–2 988 962).^1^ Most of the lives lost were men (88%) in Brazil, Colombia, El Salvador, Guatemala, Mexico, the USA, and Venezuela.^1^ The highest rate of firearm ownership is also in the USA, accounting for 40% of global ownership.^2^ Given that only 10% of firearm mortality occurs in conflict situations—a statistic that is likely to be an underestimation—this is a devastating toll from a preventable public health issue.^3^

Although these data represent the most reliable epidemiological estimates, several factors create bias towards the under-reporting of firearm injuries. For example, method-specific suicide and homicide data are only reported by a few countries, hence estimates are based on few data, inviting the potential for underestimation, especially in data-poor regions.^4^ Additionally, the causes of some firearm injuries might have been misclassified; the role of social and cultural factors that determine suicidal use of firearms are usually not well captured; and the extent to which homicide rates can be driven by illegal rather than legal ownership are poorly accounted for.^4^

In 2020, firearm-related injury became the leading cause of death in the USA among those aged 1–19 years (83% increase since 2013), surpassing motor vehicle crashes, malignant neoplasms, and drug overdose and poisoning.^5^ Although many causes of death in adolescents and young adults have declined in the past four decades, deaths from firearms have remained stagnant, partly reflecting its neglect from public health policy and its framing as a primarily electoral affair coloured by partisan politics. This inaction is reflected at the global level. The 30-year epidemiology of firearm violence across 204 countries and territories suggests little progress has been made during this time. The mortality rate from physical firearm-related violence only marginally decreased from 2·41 deaths per 100 000 people (95% CI 2·32–2·52) in 1990 to 2·29 deaths per 100 000 people (2·12–2·49) in 2019.^1^ In 2017, more than 1 billion firearms were estimated to be in global circulation, 857 million (84·6%) of which were owned by civilians.^6^ We recognise that the health impacts do not simply concern the loss of life; longer term physical and mental health consequences for survivors are also relevant, yet often unmeasured, dimensions of the firearm-related burden.

Despite this burden in young people, research agendas have not adequately aligned their funding and priority towards this area. Research funding for firearm-related death, the leading cause of death in this age group, is not commensurate with the relative harm caused, and only US$12 million is allocated for it per year, compared with $88 million allocated for motor vehicle crashes and $335 million for neoplasms.^7^ With adequate federal budgetary allocations to fund and facilitate research on firearm injury, the public health community can work collaboratively with lawmakers to assemble a robust evidence base to direct the most appropriate policy directions, and develop the case for action.

## Framing firearm injury as a public health issue

Violence is both a cause and consequence of social inequality. Structural inequalities intersect and compound the burden of firearm-related injuries. Income,^2^ poverty,^8^ underfunded public housing,^9^ under-resourced public services,^10^ underperforming schools,^10^ lack of opportunity and perceptions of hopelessness,^11^ the ease of access to firearms for high-risk individuals,^10^ and citizens’ low trust in institutions,^12^ have all been identified as factors influencing this burden. These factors play an especially prominent role in impoverished communities, where a lack of trust in institutions deemed to protect them respectfully and fairly can instil a belief that the social contract is unjust and unfit to meet their security needs, resulting in some individuals actioning the law through their own methods. These factors do not operate in silos but work in a mechanistically interconnected manner. For example, racial–ethnic segregation can influence the epidemiology of injuries by spatially constraining people of similar demographic and economic contexts to specific locations. Institutional and structural racism ushered many Black Americans to reside in hyper-segregated, low-income neighbourhoods with underfunded public services compared with their White counterparts.^13^ If governments do not allocate adequate public spending for improved social welfare and economic security to these groups, the opportunity for intergenerational social mobility is considerably reduced and the incidence of crime increases.^14^

Since 1990, upper-middle-income countries have consistently experienced the brunt of harm, with the mortality rate 2·8 times higher than the rest of the world in 2019.^1^ The epidemiological case for priority-setting is made more compelling when evaluating the spatial patterns. Across world regions, the geographical context of the firearm-related physical violence burden is exposed with great clarity: firearm injury inordinately affects people of the Americas. The death rate from firearms in the Region of the Americas was more than 13·4 times greater than the rest of the world in 2019, accounting for 68·7% of the global mortality and ranking as the twelfth leading cause of death across the region––a rank unchanged from 1990 and higher than the death rate from breast cancer, pancreatic cancer, and stomach cancer.^1^ Creating supportive environments to enable children and adolescents make social advances on their parental conditions is hampered by the damaging role of firearms in shortening life expectancy and in reducing intergenerational social mobility. In addition to the health and social consequences, the economic toll is substantial, equating to annual societal costs of more than $150 billion in the USA alone.^15^

## Evidence for effective prevention

Research on firearm-related injury is growing, from a few dozen studies published each year throughout the 1980s and 1990s to annual outputs of several hundred reports since the 2010s. The available literature paints an increasingly nuanced understanding of firearm injury and preventive implementation tools.

Relevant and effective legislation remains one of the most direct methods of tightening firearm control (panel), but often the absence of evidence-based policy making leads to several laws conferring inconsequential effects on firearm mortality. The wide differences in factors and cultures underlying firearm violence complicate the ability to identify single specific interventions that are likely to deliver the greatest benefits.^16^ Lawmakers might need to accept this degree of uncertainty and consider implementing packages of legal measures that are more likely to deliver desired results, as demonstrated successfully through the comprehensive demand–supply approach to tobacco control.^17^

Panel **Categories and subtypes of firearm-related legislation**

**Use**

• Right to carry or shall issue laws
• Hunting laws
• Stand your ground and castle doctrine laws
• Ordinances against publicly firing a gun

**Sales**

• Licensing and inspections of dealers
• Record-keeping requirements
• Background checks
• Waiting periods
• Requirement to report multiple sales
• Restrictions on number of firearm purchases
• Zoning ordinances barring gun shows on public property

**Ownership**

• Bans on purchases or possession by high-risk groups
• Licensing for owners and permits for firearms
• Required training on safe firearm use
• Requirement to notify police of stolen firearms

**Safe storage**

• Child access prevention laws
• Other safe storage requirements

**Firearms and ammunition**

• Bans on automatic and semi-automatic firearms
• Bans on high-capacity ammunition magazines

**Punishment for offenders**

• Penalties and sentences for firearm misuse

**Voluntary rendition**

• Firearm buyback programmes

Information from Santaella-Tenorio et al.^16^

Analysis shows that the three most effective laws in reducing firearm mortality in the USA are universal background checks for firearm purchases (incidence rate ratio [IRR] 0·39 [95% CI 0·23–0·67]); background checks for ammunition (IRR 0·18 [0·09–0·36]); and identification requirements either through microstamping or ballistic fingerprinting (IRR 0·16 [0·09–0·29]).^18^ Federal-level implementation of these measures could reduce mortality rates by 57% for background checks for firearm purchases, 81% for background checks for ammunition, and 83% for identification requirements.^18^ At least nine US laws have been considered ineffective and seven were not supported by evidence.^18^ For example, the *Brady Handgun Violence Prevention Act* (1993) mandated federal background checks on purchasers, but was flawed in its allowance for unlicensed dealers to freely retail firearms without any rigorous assessment.

Evidence from 130 studies in ten countries found that in some countries, the simultaneous implementation of laws targeting multiple firearms regulations reduced firearm-related mortality; and some laws specifically restricting the purchase of and access to firearms (eg, background checks and safer storage arrangements) were associated with reductions in intimate partner homicides and unintentional deaths in children.^16^ Further evidence from the USA is consistent with these conclusions, and shows that the evidence is strongest for laws strengthening background checks and laws that require a permit-to-purchase.^19^ However, the effect of many laws in current use worldwide is uncertain—for example, laws around child safety, firearm trafficking, bans on military-style assault weapons, and usage restrictions in public places.^19^

Disability-adjusted life years (DALYs) represent an important health metric for measuring the health loss from diseases and injuries across populations. Analysis of the factors contributing to the DALYs attributable to violent and suicidal uses of firearms for both sexes combined in 2019 found that alcohol use, drug use, and intimate partner violence were the most important risk factors, with high outside temperature contributing to a lesser degree.^1^ On the basis of the available evidence, we identify three measures to reduce the firearm-related injury burden that align with the so-called COM-B behaviour change framework,^20^ designed to model the US criminal law system, for which presenting evidence to prove a homicide should illustrate: means or capability; opportunity; and motive.

### Limiting access to firearms

Access to firearms and civilian firearm ownership correlate with the firearm injury burden. Although the spatiotemporal patterns of individuals affected by these injuries are important to measure, so are the spatial distributions of firearms themselves. There are more guns than people in the USA, and most deaths occur not through mass shootings but in people's homes.^21^ For cases in which individuals are found to possess one or more firearm in their homes, the risk of death doubles.^22^ Although men experience the greatest burden at the global, regional, and national levels, women are more likely to die from firearm use in their homes.

The rates of firearm injury death are greater for areas where there is more civilian access to firearms, exemplified most clearly in the cases of the USA and Venezuela.^16^ The key to reducing the ubiquity of firearms lies in interrogating the strategic influences of the firearm industry and its proponents. In assembling the evidence for optimal gun control, we should recalibrate our gaze towards the tobacco, alcohol, and nutritional industries, where countering corporate marketing and lobbying have measurably diminished their role as major commercial determinants of health. Greater accountability and oversight over the marketing and sales of guns and ammunition are also required in the firearm industry.

Designing policy during the COVID-19 pandemic demonstrated the importance of cross-country learning. The burden of firearm-related mortality in China and Japan is negligible, correlating with tedious processes for firearm procurement and bans or highly restricted permissions for civilian ownership for most major types of firearms (table). By contrast, firearms of all types (except full automatic weapons in the USA) are almost instantaneously purchasable in both the USA and Yemen, with questionable rigor associated with background checks (table). Even in some high-burden settings (Brazil, Mexico, and South Africa) where comparatively thorough procurement processes are required, the need for purchasers to evidence a specific reason for the weapons is rarely required, whereas lower-burden countries (eg, China, Japan, Germany, and Austria) require justification such as a severe personal danger or other self-defence reason (table).

**Table:** The burden of firearm-related injury, permitted types of firearms, and procurement methods in 16 countries

	**Firearm injury mortality (deaths per 100 000 people)**	**Firearm injury (DALYs per 100 000 people)**	**Firearm ownership per 100 people**	**Permitted types of firearms**				**Process of purchasing a firearm**
				Long gun	Handgun	Semi-automatic	Fully automatic	
Brazil	21·93 (21·06–22·91)	1276·56 (1224·57–1332·50)	8·29	Yes	Yes	Yes	No	Submit a statement detailing the self-defence need for a firearm; complete a course on firearm handling and demonstrate more than 60% accuracy; obtain a statement from an accredited psychologist certifying mental fitness; obtain a certificate confirming no criminal record; complete firearm purchase; register firearm with the federal police; complete an online form to transport the firearm; return to the dealer and collect the firearm
Mexico	16·31 (13·88–19·31)	892·94 (758·58–1045·29)	12·91	Yes	Yes	NA	No	Acquire a letter from the local authorities confirming absence of a criminal record; submit evidence detailing employment status and income; pass a background check (considering criminal history, employment, and current gun ownership); travel to Mexico City, where the only store authorised to sell guns is located; fingerprint identification; complete firearm purchase
South Africa	5·28 (4·26–6·96)	297·57 (232·55–392·06)	9·65	Yes	Yes	Yes	Restricted	Join an accredited shooting club, or document a need for self-defence; complete firearm safety training and pass both a written and practical assessment; present references from two employers, friends, or community leaders; fingerprint identification; pass a review (considering criminal behaviour, history of domestic violence and drug abuse, or interviews with family and neighbours); arrange firearm storage that conforms to safety regulations; allow the police to inspect firearm storage arrangement; wait several months for a federal review of the application; complete firearm purchase
USA	3·96 (3·70–4·09)	232·07 (215·60–240·48)	120·5	Yes	Yes	Yes	No	Pass an instant background check (considering criminal convictions, domestic violence record, and immigration status); complete firearm purchase
Israel	1·02 (0·93–1·11)	56·70 (51·82–62·02)	6·69	Yes	Yes	NA	No	Join a shooting club, or evidence dangerous living conditions; acquire medical approval of mental fitness and absence of drug abuse history; install appropriate firearm storage; present criminal and mental health records to the authorities; complete firearm purchase with a limited supply of bullets; demonstrate appropriate use
Yemen	0·77 (0·43–1·27)	45·86 (25·56–75·27)	52·80	Yes	Yes	Yes	Yes	Direct purchase through a firearm market or online retailer
Russia	0·78 (0·65–0·93)	39·47 (33·15–46·75)	12·29	Yes	No	No	No	Obtain a hunting license, or document the self-defence need for a firearm; complete an assessment of relevant laws, handling, and first-aid skills; acquire medical approval of mental fitness and absence of drug abuse history; attend a firearm safety and handling class and pass an assessment; apply for a license; pass a background check
India	0·57 (0·45–0·73)	29·97 (23·69–37·71)	5·30	Yes	Yes	Restricted	No	Join a shooting club, or supply proof of serious physical danger; attend a practical training course on firearm handling and shooting; obtain a medical certificate of physical and mental health; confirm appropriate firearm storage arrangements; pass a review (considering 3 years of tax returns, criminal history, mental health history and domestic violence); agree to authorities conducting interviews with family and community members; complete firearm purchase
Canada	0·47 (0·42–0·51)	28·58 (25·71–31·61)	29·99	Yes	Restricted	Yes	No	Supply proof of attendance at an approved shooting club or range, or evidence status as a collector; complete a safety course and pass both written and practical assessment; present two references; apply for a permit, and wait 28 days before processing begins; pass a background check (considering criminal record, mental health, addiction, and domestic violence history); complete firearm purchase; register purchased handguns with the police
Australia	0·18 (0·17–0·20)	11·42 (10·42–12·50)	14·50	Restricted	Restricted	Restricted	No	Join and regularly attend a shooting club, or evidence status as a collector; complete a course on firearm safety and operation, and pass both a written and practical assessment; arrange firearm storage that conforms to safety regulations; pass a review (considering criminal history, domestic violence, restraining orders, and arrest history); agree to authorities conducting interviews with family and community members; apply for a permit to acquire a specific type of firearm; wait for a minimum 28 days; complete firearm purchase for the specific type of firearm for which the permit was received
New Zealand	0·15 (0·14–0·16)	8·90 (8·27–9·60)	26·32	Restricted	Restricted	Restricted	No	Pass a background check (considering criminal convictions, domestic violence record, medical status, and mental health); present character references; pass an in-person home security inspection with assessment of storage arrangements; complete a firearm safety course; seek approval for a firearms license; complete firearm purchase
Austria	0·15 (0·14–0·16)	8·57 (7·74–9·54)	30·00	Restricted	Yes	Restricted	Restricted	Supply proof of serious physical danger; pass a criminal history review; complete a mental health survey, and complete a psychological and physical test; complete a course on safe firearm handling and storage; install safe storage; complete firearm purchase
Germany	0·10 (0·09–0·11)	6·19 (5·48–6·99)	19·62	Yes	Yes	Yes	No	Join a shooting club, acquire a hunting license, supply proof of serious physical danger, or evidence status as a collector; demonstrate specialised knowledge of firearms, which might involve a written assessment and practical demonstration of safe handling; if younger than 25 years, submit a medical certificate of mental fitness; confirm appropriate firearm storage arrangements; pass a background check (considering criminal history, mental health, and drug addiction); apply for a permit to purchase a specific gun; complete an additional short background review; complete firearm purchase
UK	0·04 (0·04–0·04)	3·81 (3·24–4·49)	4·64	Restricted	Restricted	No	No	Acquire a firearms certificate; present references; pass an in-person background check (including an in-person home police interview with assessment of storage arrangements); complete firearm purchase
Japan	0·02 (0·02–0·02)	2·26 (1·79–2·87)	0·30	Restricted	No	No	No	Complete a firearm course and pass a written examination; seek medical confirmation of mental fitness and evidence absence of a drug abuse history; apply for a permit for firearm training; justify the reason for firearm need via a police interview; pass a review (considering criminal convictions, firearm possession record, employment, involvement with organised crime groups, personal debt, and relationships with friends and family or neighbours); apply for a gunpowder permit; complete a training class and pass a firing test; obtain a certificate from a firearm dealer describing the desired firearm; apply for a hunting license, if intended for this use; purchase a firearm safe and ammunition locker, conforming to safety regulations; allow the police to inspect firearm storage arrangement; pass an additional background review; complete firearm purchase
China	0·02 (0·02–0·03)	2·37 (1·93–2·93)	3·58	Restricted	No	No	No	Establish a specific reason for firearm possession; arrange for firearm storage at a gun range, remote hunting ground or pastoral area; demonstrate knowledge of safe gun use and storage; pass a background check (considering mental illness, criminal record, and domestic violence); complete firearm purchase

Countries are listed in ascending order by the rate of DALYs from firearm-related injury through physical violence in 2019 per 100 000 people. Epidemiological estimates were extracted from the Global Burden of Disease Study 2019 produced by the Institute for Health Metrics and Evaluation,^1^ data on civilian firearm holdings were extracted from the Small Arms Survey in 2018,^6^ permitted types of firearms were extracted through literature searches, and methods of international firearm procurement were adapted from analysis by the New York Times.^23^ DALYs=Disability-adjusted life years.

In high-burden settings, there exists the more arduous challenge of establishing a culture of firearm safety. Our literature searches revealed that research informing evidence-based strategies to address the number of firearms in current circulation are lacking in the academic discourse. This understanding is crucial to prevent aversion to political action due to its perceived futility. Decision makers might be encouraged by a phased approach to limiting access, where targeting young people could forge a new culture around gun safety and pay dividends over a longer period. These patterns have been observed in the burden of tobacco smoking: although population growth has increased the total number of smokers globally since 1990, the prevalence of smoking has steadily decreased in this period.^24^, ^25^ Responsible firearm dealers and owners should be involved in solutions, such as insisting mandatory training and licensing, and requiring safe storage arrangements.

### Reducing alcohol use

Alcohol use has been identified as the most prominent risk factor contributing towards the health loss from violent (23 525 deaths attributable to alcohol use) and suicidal (10 069 deaths) uses of firearms, while occupational injuries are a more relevant attributable risk factor to the health loss caused by unintentional firearm injuries.^1^

Due to the role of alcohol in reducing inhibitions and increasing confidence, some individuals attempting suicide might use alcohol to overcome their apprehensions or fear.^26^ Evidence suggests that intoxication might not be a direct cause of death by suicide, but the ease of access to alcohol and other substances might contribute to death by suicide.^26^, ^27^

The costs accumulated through additional health-care delivery, increased crime, and lost productivity due to alcohol and illicit drug use exceed $400 billion annually.^28^ In addition to firearm control policies, greater restrictions on alcohol are associated with decreased rates and odds of self-harm and suicide from firearms; this protective relationship is most effective for suicides involving both alcohol and firearms.^29^ Secondary effects through tighter alcohol control are beneficial to health systems, and the WHO list of so-called best buys are evidence-based recommendations to better control alcohol use and other non-communicable diseases and risk factors.^30^

### Preventing intimate partner violence

Men are the most likely perpetrators of firearm violence, often in the context of domestic and relationship violence.^4^ Addressing intimate partner violence––the most common form of violence globally––could have averted 5450 firearm homicides in 2019.^1^ Gender-based violence should garner further priority in post-pandemic public health, as the number of women reporting an increase in such violence rose by nearly a quarter since the acute phase of the pandemic, reversing the little existing progress.^31^ A compelling aspect of these interventions is their potential to provide synergistic benefits. There is a strong combination prevention case for efforts to reduce firearm and intimate partner violence, particularly when viewed through a global development lens, in which eliminating violence against women and girls is a target for Sustainable Development Goal (SDG) 5––achieve gender equality and empower all women and girls.

The correlation between intimate partner violence and firearm injury is well known in criminal justice systems. In the USA, convicts of a qualifying misdemeanour crime of domestic violence are prohibited from possessing firearms or ammunition.^32^ However, there are several exceptions to this law that grant a wide number of convicts exemption.^32^

Intimate partner violence is associated with mental disorders, particularly self-harm and suicidality. Adults in England reporting a lifetime history of intimate partner violence were found to be three times more likely to have made a suicide attempt (adjusted odds ratio [OR] 2·82 [95% CI 1·54–5·17]) and twice as likely to have self-harmed (OR 2·20 [1·37–3·53]) or had suicidal thoughts (OR 1·85 [1·39–2·46]) than those who had not reported having experienced intimate partner violence.^33^ Readily accessible firearms, a history of intimate partner violence, and alcohol use can create the conditions that amplify this burden.

The mental health dimensions of gun control––while highly relevant to public health in its broader sense––are unlikely to translate into substantial improvements in the burden of firearm injury, other than through secondary benefits in preventing intimate partner violence. Furthermore, encouraging a political hyperfocus on enhanced mental health care might distract from other, more direct solutions forecasted to deliver greater effects, such as weapons bans (eg, automatic discharge firearms), smart firearms (eg, discharge only with owner's fingerprints), safe storage arrangements, training, and thorough background checks.^17^ Additionally, no clear relationship exists between mental health and firearm homicides,^34^ hence the ambition of some US policy makers to primarily target mental health to prevent mass shootings does not align with the epidemiological evidence.

## Conclusion

With adequate political prioritisation and comprehensive firearm control efforts, the cycle of grief, anger, activism, deflection, and inaction can be escaped, and a major cause of avoidable deaths can be averted globally. To disallow the political opportunity for these cycles to revolve, we should recognise the power of social mobilisation and learn from the many successes across global health and harness the vast potential of civil society. Fostering a rich collaboration between health, criminal justice, security, faith, education, and civil society is central to promoting sustainable social change, transitioning the cultural consensus away from violence-supportive norms and towards peace and equality. We should sharpen our anger for the millions of adolescent and young adult lives lost and those who live at risk from this grossly preventable cause of death. The ambition of SDG 16––promote peaceful and inclusive societies––where the mandate to substantially reduce all forms of violence and related deaths can be found, will not be achieved by 2030 without courageous leadership and meaningful action devoid of partisan politics.

### Search strategy and selection criteria

References for this Viewpoint were identified by searching the PubMed database for articles published between Oct 1, 1997, and May 1, 2022. The search strategy was also complemented by Scopus and Google Scholar searches with no date restrictions. We used the keywords “firearm*”, “gun*”, “homicid*“, injur*”, “burden”, “health” and “violen*”. Our search included articles and reviews published in English. Reference lists of relevant articles were also screened and hand-searching broadened the search. The final reference list was generated on the basis of originality and relevance to the broad scope of this Viewpoint. The majority of the epidemiological data were obtained via the Institute for Health Metrics and Evaluation Global Burden of Disease Compare tool.

## Declaration of interests

We declare no competing interests.
